# Enhanced Untargeted Metabolomics Based on High-Resolution Mass Spectrometry Reveals Global Rewiring Due to Mitochondrial Dysfunction in Yeast

**DOI:** 10.3390/ijms27062624

**Published:** 2026-03-13

**Authors:** Fabrizio Mastrorocco, Luca De Martino, Igor Fochi, Graziano Pesole, Ernesto Picardi, Clara Musicco, Sergio Giannattasio

**Affiliations:** 1 Institute of Biomembranes, Bioenergetics and Molecular Biotechnologies, National Research Council of Italy, 70126 Bari, Italy; fabrizio.mastrorocco@cnr.it (F.M.); l.demartino@ibiom.cnr.it (L.D.M.); graziano.pesole@uniba.it (G.P.); ernesto.picardi@uniba.it (E.P.); clara.musicco@cnr.it (C.M.); 2 Istituto Zooprofilattico Sperimentale dell’Abruzzo e del Molise G. Caporale, 64100 Teramo, Italy; i.fochi@izs.it; 3 Department of Biosciences, Biotechnologies and Environment, University of Bari “Aldo Moro”, 70125 Bari, Italy

**Keywords:** untargeted metabolomics, mass spectrometry, metabolite annotation, yeast, mitochondrial dysfunction, mitochondrial retrograde pathway

## Abstract

Mitochondrial dysfunction profoundly alters cellular metabolism, yet its systems-level consequences remain incompletely characterized. Here, we present a comprehensive untargeted metabolomics analysis of respiratory-deficient (ρ^0^) and competent (ρ^+^) *Saccharomyces cerevisiae* prototrophic cells using ultra-high-performance liquid chromatography coupled to Orbitrap Fusion™ Tribrid™ high-resolution mass spectrometry. By integrating hydrophilic interaction and reversed-phase chromatography in both ionization modes, we detected ~7000 features per chromatographic condition, of which ~12% were structurally annotated through MS^n^ fragmentation and in silico spectral matching. Principal component analysis revealed distinct metabolic signatures between ρ^0^ and ρ^+^ cells, with ~73% of total variance explained by the first two components. Volcano plot and hierarchical clustering analyses identified a marked accumulation of phosphate-containing metabolites, sphingolipids, ceramides, and fatty acid residues in ρ^0^ cells, whereas amino acids, excluding arginine, cysteine, and aromatics, were enriched in ρ^+^ cells. Notably, branched-chain amino acid depletion in ρ^0^ cells correlated with impaired growth and mitochondrial stress. Pathway enrichment analysis, supported by transcriptomic integration, prompted us to further investigate reprogramming of polyamine biosynthesis and aromatic amino acid metabolism. Calibration curves constructed from certified standards validated the accuracy of the LC–MS platform and reinforced annotation confidence. Our findings demonstrate that advanced untargeted metabolomics, coupled with MS^3^ fragmentation and multi-omics integration, enables high-resolution mapping of metabolic reconfiguration under mitochondrial dysfunction, offering mechanistic insights into mitochondrial retrograde signaling and adaptation.

## 1. Introduction

The full complement of low molecular weight compounds (usually <1500 Da), present within a biological system either as natural metabolites or derived from environmental sources, constitutes its metabolome. The metabolome is the product of both intracellular processes and chemical–physical environmental conditions, reflecting the complex interaction between genome and environment, i.e., the phenome, which defines the individuality of an organism or biological system. The phenome comprises multiple layers of phenotypes, including but not limited to genome, transcriptome, and proteome, resulting from intricate interactions among these layers [1]. While the genome, transcriptome, and proteome profoundly influence the metabolome, the metabolic background itself exerts a pivotal effect on gene expression epistasis in cells and organisms [2]. Accordingly, metabolomics represents the deepest and most unifying layer within the phenome in a systems biology framework [3].

Metabolomics analysis enables the determination and study of the metabolome and requires a diversity of analytical methodologies within a multidisciplinary approach. Commonly employed techniques include liquid chromatography mass spectrometry (LC-MS), gas chromatography mass spectrometry (GC-MS), capillary electrophoresis mass spectrometry (CE-MS), nuclear magnetic resonance (NMR) spectroscopy, and imaging mass spectrometry [4]. Metabolomics data, alone or integrated with genomics and proteomics, can serve as biomarkers that illuminate the underlying characteristics and complexities of biological systems [5].

Metabolomics analysis may be either targeted or untargeted. Targeted approaches allow the quantification of a defined set of molecules involved in specific metabolic pathways and/or xenobiotic metabolism. Untargeted approaches, in contrast, aim at the comprehensive analysis of all measurable compounds in a sample, thereby enabling the determination of the metabotype (metabolic phenotype) of a biological system. However, no complete experimental characterization of any organismal or cellular metabolome has been achieved to date, in sharp contrast to the diversity of organisms whose genomes have been fully sequenced. This discrepancy arises from the vast chemical diversity of small molecules, which, unlike genomics with only four nucleotides and finite combinations, encompass an almost infinite chemical space [6].

Despite the increased availability of high-resolution (HR) mass spectrometry systems, confirmed structural identification of all detected compounds remains a formidable challenge. A typical metabolomics workflow comprises sampling, metabolite detection by MS and/or NMR spectroscopy, data processing, and statistical analysis to identify peaks of interest. Annotation of metabolomics data is often manual, and the identification of peaks absent from databases is a time-consuming and rarely performed step [7]. The field is rapidly evolving as new analytical strategies and computational algorithms are developed [8,9]. To improve both quantitative and qualitative aspects of metabolomics databases, a confidence-level system has been introduced for annotation, classifying detected structures as confirmed or probable (levels 1 and 2), tentative candidates (level 3), or unknown exact masses (levels 4–5) [10,11,12]. An ultra-high-performance liquid chromatography (UHPLC) system coupled to a high-resolution mass spectrometer (HRMS), based on FT-orbital trap, with MS^n^ capabilities, were employed as analytical platform. Following acquisition, raw data files were processed in order to perform peak alignment and area normalization. It features filtering background signals, interrogating MS^n^ spectral libraries for compound annotation, and conducting statistical analyses to evaluate relative differences in metabolite accumulation across samples [13].

As analytical efficacy increases, so does the demand for enhancing untargeted metabolomics analysis using state-of-the-art systems. It has been recognized that advancing metabolome characterization should initially focus on a few model organisms, leveraging the critical mass of research activity and knowledge surrounding them. Once their metabolomes are successfully identified, this knowledge will be of great value to the scientific community [14].

Yeast has emerged as a powerful model organism for systems biology and integrative omics analysis in both fundamental and applied research. It provides a suitable platform for developing genome-scale models (GEMs), which are mathematical representations of a cell modeling the interplay of metabolites, reactions, and genes [15]. The so-called “petite yeasts” (ρ^0^), characterized by complete loss of mitochondrial DNA (mtDNA), are unable to grow on media containing only non-fermentable carbon sources (e.g., ethanol or glycerol) and have been instrumental in studying mitochondrial dysfunction [16], a key factor in the molecular etiology of human diseases [17]. Yeast can survive mtDNA loss, albeit with reduced growth rate [18], through an adaptation mechanism triggered by the mitochondrial retrograde response [16].

In systems biology, comprehensive physiological studies require the generation and analysis of multi-omics datasets for the same model organism, typically achieved through multilaboratory efforts. Within this framework, the Yeast Systems Biology Network (YSBN) generated prototrophic diploid *Saccharomyces cerevisiae* strains, YSBN1 and YSBN2, as direct derivatives of S288c [19,20]. Using YSBN1, it was demonstrated that the slow growth of ρ^0^ cells results from partial auxotrophy in four amino acids due to altered iron metabolism and tricarboxylic acid cycle inhibition [18]. However, a comprehensive understanding of metabolic reprogramming induced by mtDNA depletion and ensuing dysfunction remains incomplete.

Here, we used this specific UHPLC–HRMS platform to enhance metabolite identification and annotation in untargeted metabolomics analysis, thereby getting insights into mitochondrial dysfunction using a yeast model system.

To test efficiency and accuracy of untargeted metabolomics analysis with state-of-the-art equipment, we performed a broad-spectrum comparative untargeted metabolomics study of ρ^0^ and ρ^+^ YSBN1 prototrophic cells using metabolomics HRMS platform [13,21]. A single extraction method was employed to maximize information recovery without specific extractions for each metabolite class. By combining hydrophilic interaction chromatography (HILIC) and reversed-phase (RP) C18 separations, each acquired in both positive and negative ionization modes, we detected approximately 7000 features (compounds) after background subtraction. Of these, approximately 12% were confidently annotated based on MS^2^ and MS^3^ fragmentation data, a proportion that exceeds the typical annotation rates reported in the literature [10].

## 2. Results

### 2.1. Experimental Quality Control Evaluation

System stability was evaluated by two complementary approaches: (i) comparison of total ion chromatograms (TICs) from quality control (QC) samples (Unlabeled Metabolite Yeast Extract, Cambridge Isotope Laboratories, Inc., Tewksbury, MA, USA), and (ii) statistical assessment of sample reproducibility across the four chromatographic methods using principal component analysis (PCA).

QC TICs obtained in both positive and negative ionization modes for HILIC and RP-C18 separations showed highly comparable retention times and response intensities, with minimal instrumental drift. Internal standards (3-Acetylindole, L-Acetyl N-Phenylalanine) exhibited stable retention times and peak intensities across all runs, and no evidence of carry-over or cross-contamination was detected. These results confirm the robustness of the LC–MS method and the reliability of data acquisition. The stability of the QC samples and the consistent number of injected cells allowed us to avoid software-based area normalization.

### 2.2. Analysis of ρ^0^ vs. ρ^+^ Cells Metabolome

**Figure 1:**
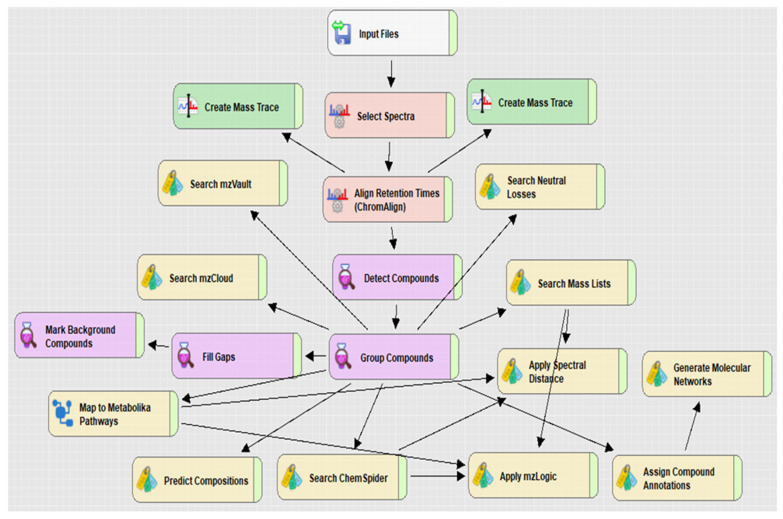
Workflow from Compound Discoverer 3.3. The data processing nodes and associated workflow connections used for processing raw data are shown.

Untargeted metabolomics analysis was performed on exponentially growing ρ^+^ and ρ^0^ YSBN1 cells using Compound Discoverer™ (CD) software (workflow in Figure 1). Each workflow box represents a node within which parameters can be customized and configured to enhance the accuracy and efficiency of metabolite annotation. Critical components of the workflow include nodes such as ChemSpider, which allows the integration of external databases for compound identification, and Metabolika, which facilitates the mapping of annotated metabolites onto metabolic pathways.

**Figure 2:**
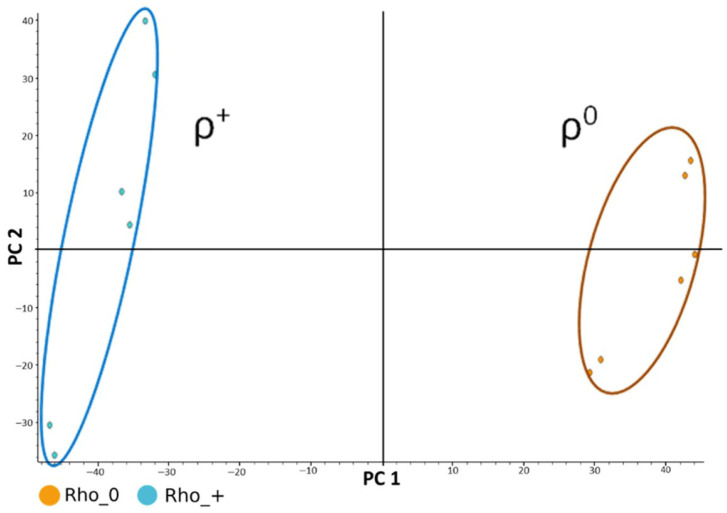
Principal Component Analysis (PCA), highlighting two clusters: the ρ^+^ samples on the left (light blue) and the ρ^0^ samples on the right (orange).

PCA revealed clear separation between ρ^+^ and ρ^0^ samples across all chromatographic modes (Figure 2), ρ ^+^ samples on the left (shown in light blue) and the ρ^0^ samples on the right (shown in orange). Each sample includes three biological replicates and two technical replicates, which are clearly distinguishable. The first two principal components, PC1 and PC2, together explain approximately 73% of the total variance in the dataset. Biological variability accounted for ~18% in ρ^+^ samples and slightly less in ρ^0^, while instrumental variability was ~5%. To avoid features with larger absolute areas dominating the analysis, peak areas were log-transformed as log (x + 1), where x is the peak area, prior to multivariate analysis.

To provide a formal statistical confirmation of the visual separation, we performed a one-way PERMANOVA on the full dataset (Distance: Euclidean, Permutation N: 9999, *p*-value (Bonferroni): 0.0316). This analysis demonstrated a highly significant effect of biological condition.

**Figure 3:**
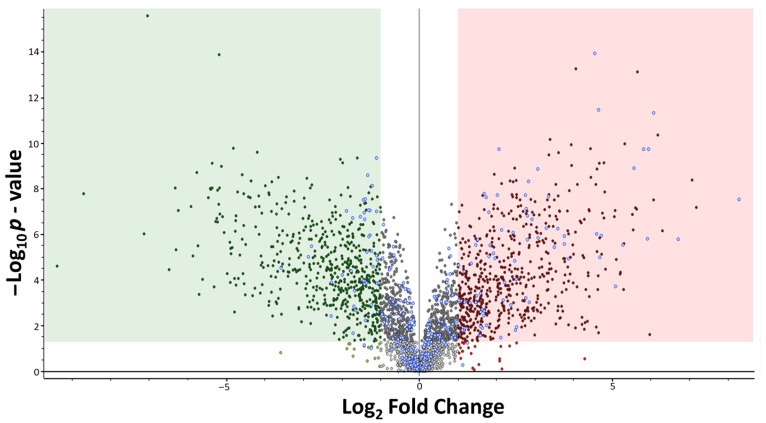
The volcano plot displays all detected metabolites. The red and green regions satisfy the conditions of an adjusted *p*-value ≤ 0.05 (Benjamini–Hochberg correction) and a log2 fold change greater than 1 or less than −1 respectively. Light grey dots are unannotated metabolites with adjusted *p*-value > 0.05. Dark grey dots are unannotated metabolites with adjusted *p*-value ≤ 0.05 but −1 ≤ log2 fold change ≤ 1. Blue-grey dots are metabolites that were successfully annotated.

Volcano plot analysis identified metabolites with statistically significant differences between ρ^0^ versus ρ^+^ yeast cells (Figure 3). Identified metabolites are shown as empty blue circles, while unknown features are indicated in red/green. Metabolites enclosed within the green and red regions exhibit a statistically significant variation, defined by a *p*-value ≤ 0.05 and a log2 fold change greater than 1 or less than −1. Metabolites located in the upper right quadrant are more abundant in the ρ^0^ cluster, whereas those in the upper left quadrant are more abundant in the ρ^+^ cluster.

**Figure 4:**
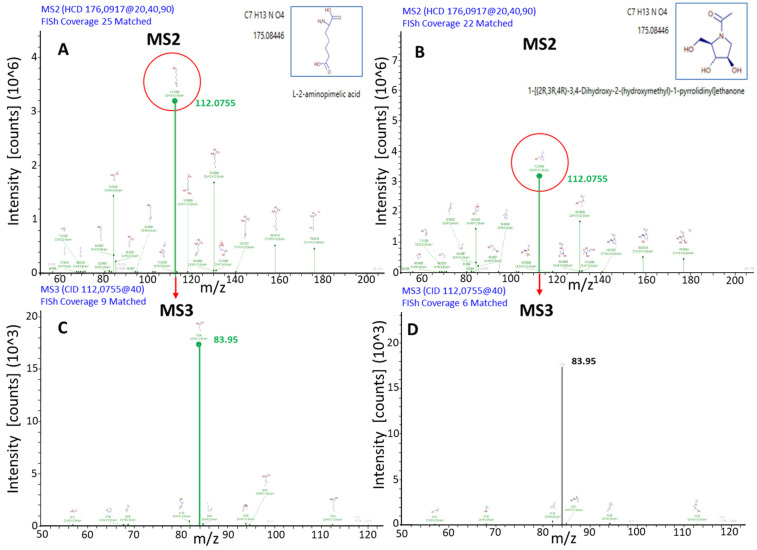
The figure shows the MS^2^ fragmentation spectra (**A**,**B**) and the MS^3^ fragmentation spectra (**C**,**D**) of the 175.08446 Da ion. The observed fragment ions matching to reference MS^2^ and MS^3^ spectra are in green.

Metabolite annotation was performed using CD-embedded databases (KEGG, BioCyc, ChEBI, LipidMAPS), online spectral libraries (mzCloud) and in-house developed RT-annotated spectral libraries (mzVault™). Overall, ~1800 features per chromatographic mode were obtained after background subtraction, of which 10% could be structurally automatic annotated, consistent with previous reports [10,22]. Structural assignment was refined using the FISh fragmentation tool, which compares experimental MS^n^ spectra with in silico fragmentation patterns [10,11]. For example, annotation of a feature at *m*/*z* 175.08446 required MS^3^ spectra to discriminate between two isobaric candidates (Figure 4). The upper panel (Figure 4A,B) displays the MS^2^ fragmentation spectra of an ion with M.W. = 175.08446 Da, along with the corresponding in silico fragment matches generated using the FISh tool. No significant differences are observed between the two MS^2^ spectra of putative structures shown in Figure 4A,B. This is because the two spectra exhibit essentially the same fragment-ion coverage and show an almost identical match of the major fragment peaks. However, the MS^3^ spectra in Figure 4C,D—resulting from the fragmentation of the ion at *m*/*z* = 112.07550—reveal a clear distinction: only the putative structure on the left exhibits a higher spectral match compared to the one on the right. The MS^3^ spectrum of ion 112.0755 in Figure 4C shows a greater coverage of the main fragmentation peak compared with the corresponding spectrum in Figure 4D. This evidence supports the left-hand structure as the more probable isomeric candidate for the ion at M.W. = 175.08446 Da. This demonstrates the added value of MS^3^ fragmentation in resolving ambiguous structures, an innovation that expands coverage of the “dark metabolome” [10,12].

Annotation confidence was classified according to established guidelines [11,12]: Level A: identified metabolites confirmed by pure standards; Level B(i): putatively annotated compounds with full spectral match; Level B(ii): putatively characterized compounds with partial match and FISh support; Level C: unknown features defined only by accurate mass (Table S1).

Using this approach, 38 new compounds were confidently annotated (Table S1), corresponding to ~4.5% of the total annotated features (Annotation level B(ii)).

In metabolomics, the highest confidence level of annotation (Level A) can only be achieved through direct comparison with certified chemical standards [10,11]. To illustrate this principle, we selected six metabolites (Figure S1) that had shown full concordance across all queried databases and libraries (KEGG, BioCyc, ChEBI, LipidMAPS, and mzVault™, mzCloud), thereby representing robust candidates for annotation. Calibration curves (Figure S1) were generated for six representative metabolites to validate the quantitative accuracy of the LC–MS platform. Although chosen as examples, these metabolites were treated as verified standards to demonstrate that compounds with complete database agreement can be considered equivalent to Level A annotations by analogy with chemically validated references.

The selected metabolites are highlighted in violet in Supplementary Table S1. Calibration curves were constructed to assess the linearity of instrumental response within the concentration ranges typically observed in our yeast extracts. Each metabolite exhibited excellent linearity across the tested range, confirming that the LC–MS system provides reliable quantitative measurements under the applied conditions. This validation step not only strengthens the confidence in annotation for these specific metabolites but also supports the broader assumption that full database concordance, when combined with calibration-based verification, can serve as a proxy for Level A annotation in untargeted metabolomics workflows.

### 2.3. Metabolomic Profile Reconfiguration in ρ^0^ Cells

**Figure 5:**
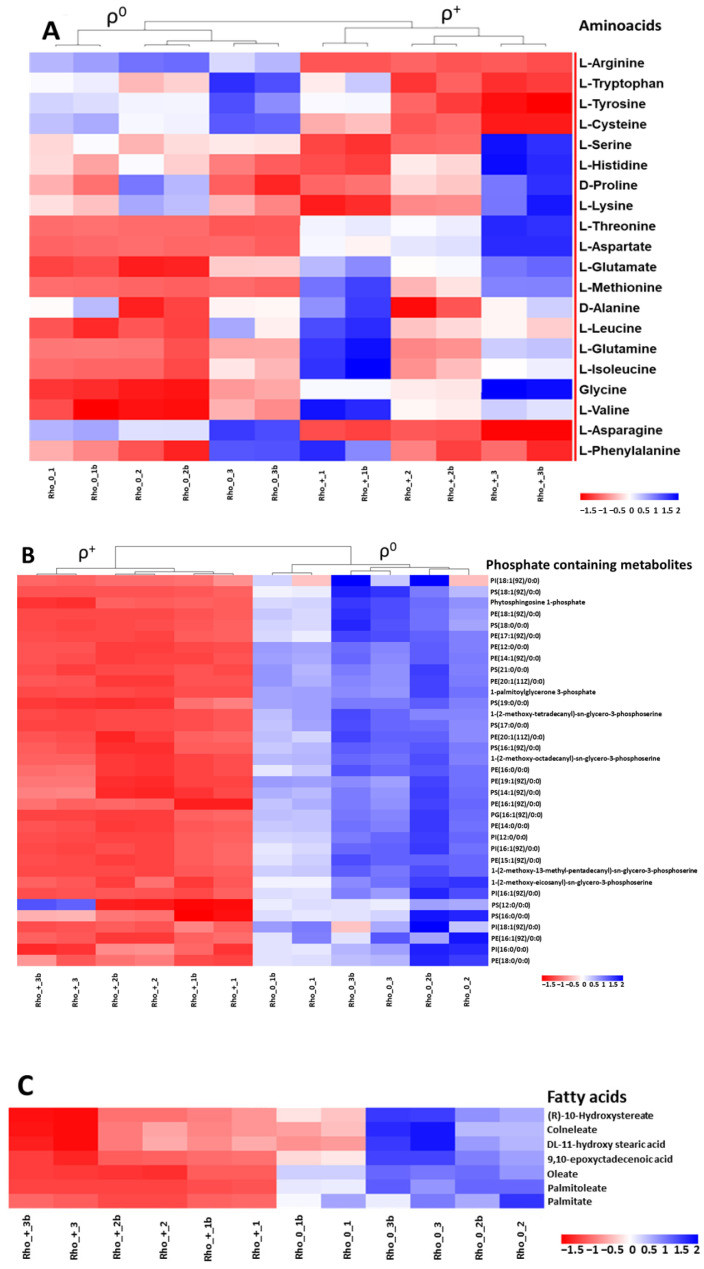
Heatmaps of amino acids (**A**) and phosphate-containing metabolites (**B**), sphingomyelins, ceramides, and fatty acids (FA) (**C**). Low concentration of metabolites in red, high concentration of metabolites in blue, indicating levels above the average abundance for each metabolite. We analyzed three biological replicates for ρ^+^ and ρ^0^ cells; each MS analysis included two technical replicates. Sample labels: Rho+ = ρ^+^; Rho0 = ρ^0^; 1–3 = biological replicates; b = second technical replicate.

Hierarchical clustering and heatmap image (Figure 5A,B) reveal a preferential accumulation of phosphate-containing metabolites, sphingomyelins, ceramides, and fatty acid moieties in ρ^0^ cells. In contrast, amino acids are generally more abundant in ρ^+^ cells, with notable exceptions including arginine, cysteine, and aromatic amino acids, which do not follow this trend. The observed depletion of branched-chain amino acids (BCAAs) in ρ^0^ cells aligns with their compromised proliferative capacity under respiratory-deficient conditions [21].

Using the CD Metabolika tool, the metabolic pathways associated with significantly altered compounds were reconstructed. Dozens of pathways obtained through CD Metabolika were analyzed and interconnected to generate a comprehensive overview of the metabolic reconfiguration occurring in respiratory-deficient yeast. Among all reconstructed pathways, polyamine biosynthesis and aromatic amino acid metabolism were selected for detailed inspection because they contained the highest number of differentially expressed metabolites and showed the strongest biological relevance to mitochondrial dysfunction.

Polyamine accumulation is a known marker of mitochondrial stress [17], while increased aromatic amino acid intermediates suggest a compensatory reprogramming of nitrogen metabolism. The imbalance between elevated aromatic amino acids and reduced BCAAs reflects a broader metabolic rewiring associated with mitochondrial dysfunction.

**Figure 6:**
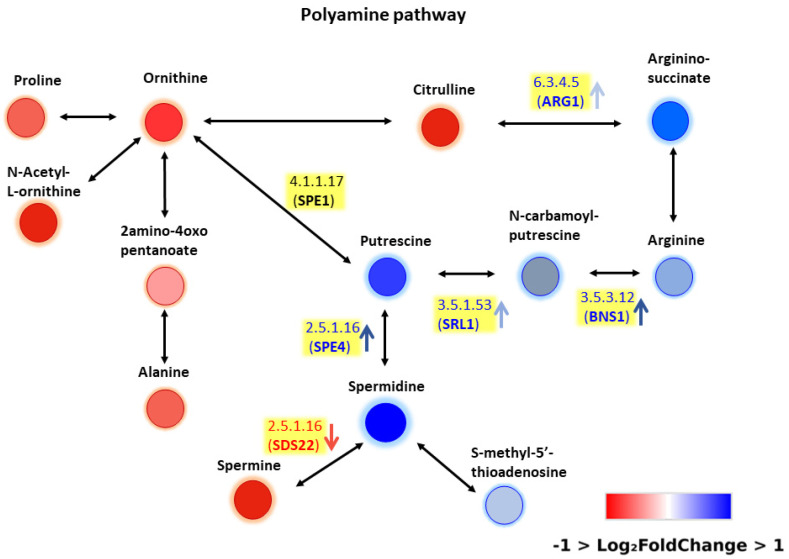
Polyamine metabolic pathway. Upregulated metabolites and enzyme transcripts are indicated in blue, downregulated ones in red.

**Figure 7:**
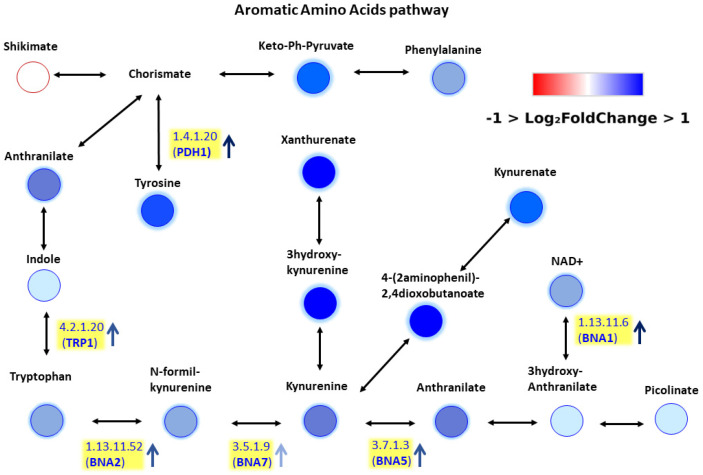
Metabolic pathway of aromatic amino acids. Upregulated metabolites and enzyme transcripts are indicated in blue, downregulated ones in red.

Based on these metabolomics data we decided to perform a pathway enrichment. Our aim was twofold: to get a first insight into the pathway involvement of metabolites altered in mtDNA-depleted cells and to test the widely used open-source tool MetaboAnalyst 6.0 [23]. Thus, we manually integrated our metabolomic data with transcriptomic data previously reported by Epstein et al. (2000) [24], which provided gene expression profiles during ρ^+^ and ρ^0^ yeast cell growth. To the best of our knowledge this study is so far from the best characterized metabolic reprogramming profile of mtDNA-depleted yeast cells based on whole genome expression profile. For each metabolite and intermediate, the corresponding enzymes were identified, and the genes encoding these enzymes were retrieved from the *Saccharomyces cerevisiae* Genome Database (SGD) (Figure 6 and Figure 7). Overall, the integration of metabolomic and transcriptomic data revealed a consistent pattern, with changes in metabolite levels generally reflecting corresponding alterations in the expression of their associated genes, supporting a coordinated reprogramming of polyamine biosynthesis and aromatic amino acid metabolism in response to mitochondrial dysfunction.

Because manual integration is inherently subjective and not fully reproducible, we adopted an automated approach using the open-source tool MetaboAnalyst 6.0. Specifically, the Joint Pathway Analysis module was used by uploading the lists of differentially expressed genes and metabolites, and the most significant pathways identified are reported in Table 1. This automated integration provides a reproducible workflow, but it also has limitations: for example, the polyamine biosynthesis pathway was not identified as a separate entity, since MetaboAnalyst relies on KEGG-based generic pathway definitions.

In this framework, polyamine metabolism is included within the broader arginine and proline metabolism pathway (Figure S2), which dilutes the statistical signal and lowers the −log10 (*p*-value), as only one subset of the pathway is affected. These findings highlight that, while automated tools improve reproducibility, manual curation remains necessary to accurately capture yeast-specific biological context and fully interpret multi-omics data.

By combining metabolomic and transcriptomic information, we obtained a putative picture of metabolic reprogramming under respiratory dysfunction. Two pathways emerged as particularly relevant: polyamine biosynthesis (Figure 6) and aromatic amino acid metabolism (Figure 7). Polyamine accumulation is a well-established marker of mitochondrial stress [17], while the accumulation of aromatic amino acid intermediates is consistent with elevated levels of these metabolites in ρ^0^ cells. Epstein et al. have shown that respiratory deficiency induces a suite of genes associated with both peroxisomal biogenesis and activities and metabolite restoration (anaplerotic) pathways that would mitigate the loss of a complete tricarboxylic acid cycle [24]. Our metabolomics and transcriptomics data integration analysis suggests a more complex picture of metabolic reprogramming in ρ^0^ cells. In order to confirm the role of polyamine and aromatic amino acid metabolism in response to mitochondrial dysfunction, further experimental evidence must be gathered including combined differential metabolomics and transcriptomics analysis as well as different cell growth phases.

## 3. Discussion

This study demonstrates that untargeted metabolomics, combined with advanced MS^3^ fragmentation and in silico annotation tools, provides novel insights into the metabolic consequences of mitochondrial DNA depletion in yeast.

The integration of MS^3^ fragmentation spectra with FISh-based in silico fragmentation significantly improved annotation confidence, enabling discrimination of isobaric metabolites that remain unresolved with MS^2^ alone. This approach increased annotation coverage by ~4.5%, consistent with recent advances in metabolomics workflows [10,21]. Such methodological improvements are crucial for addressing the “dark matter” of metabolism, i.e., the large fraction of features detected but not structurally identified [13].

Respiratory-deficient (ρ^0^) yeast cells exhibited profound metabolic reprogramming. The accumulation of phosphate-containing metabolites and sphingolipids suggests altered energy and membrane homeostasis. Reduced BCAA levels are consistent with impaired growth and reflect disrupted mitochondrial anaplerotic fluxes [21]. Conversely, elevated aromatic amino acids and polyamines indicate compensatory pathways activated under mitochondrial stress [17].

Our differential metabolomics data (Figure 5 and Figure S2) align with previous reports of mitochondrial retrograde signaling and metabolic rewiring in yeast. The observed imbalance between amino acid classes highlights the central role of mitochondria in coordinating nitrogen metabolism and stress adaptation [15,16,21].

Our results highlight the importance of integrating metabolomics with genomics and/or proteomics to fully capture the systems-level consequences of mitochondrial dysfunction. Despite morphological and cellular differences, the high degree of homology between several thousand yeast and human genes together with the deep conservation of cellular pathways between yeast and humans, such as AMPK/Snf1 and TORC1/Tor1 signaling, make yeast a preferred model organism in biomedical research [25]. Much of our current understanding of molecular cell biology, including mitochondrial function and dysfunction, comes from studies in the *Saccharomyces cerevisiae*, which remains a powerful model for dissecting these processes, with direct relevance to human pathologies involving mitochondrial defects [17,26,27]. Moreover, many human genes can be successfully substituted for their yeast equivalents and sustain yeast growth. Hence, “humanized yeast” permits measuring human protein activity in a simplified organismal context [28,29]. Thus, the development of a yeast systems biology-based multiomics integrative workflow will foster the elucidation of the molecular basis of human diseases in a personalized medicine perspective.

Future work should expand annotation coverage through integration of machine learning approaches [11,12] and standardized extraction protocols [21,23], while focusing on quantitative flux analyses to validate pathway-level reprogramming.

Current literature often treats multi-omics integration as a mere combination of datasets, without adequately considering biological context or species-specific features [30]. Our study highlights this limitation: while automated tools such as MetaboAnalyst 6.0 provide useful pathway-level insights, the outputs are based on KEGG pathway definitions and are not specifically tailored to yeast. Consequently, although MetaboAnalyst 6.0 is a valuable tool for rapid, reproducible pathway screening, mechanistic conclusions for *Saccharomyces cerevisiae* require careful manual curation and validation.

## 4. Materials and Methods

### 4.1. Standards and Solvents

A multi-metabolite standard (Unlabeled Metabolite Yeast Extract) was purchased from Cambridge Isotope Laboratories, Inc. and dissolved in the appropriate solvent following the manufacturer’s instructions. This standard was used to build an in-house library (internal mzVault library) of yeast and was run as a Quality Control (CIL-QC) in LC-MS analysis. The LC-MS grade solvents utilized in this study were as follows: LC-MS Chromasolv™ Acetonitrile (ACN) (Riedel-de-Haën™), LC-MS Chromasolv™ Methanol (MeOH) (Riedel-de-Haën™), LiChropur™ Ammonium formate (NH_4_HCO_2_) (SIGMA-ALDRICH) and Optima Formic Acid (FAc) (Fisher Chemical, Waltham, MA, USA).

### 4.2. Yeast Cell Growth

Experiments were performed with wild-type (ρ^+^) YSBN1, a prototrophic diploid derivative of *Saccharomyces cerevisiae* S288c [19,20]. Cells depleted of mitochondrial DNA (ρ^0^) were generated by treating yeast cultures with 0.1 g L^−1^ ethidium bromide in YPD medium, followed by incubation at 30 °C for 48 h. The absence of mtDNA was confirmed in isolated clones by DAPI staining and by the lack of growth on glycerol-containing selective plates. Media were solidified with 2% agar (DIFCO™).

**Figure 8:**
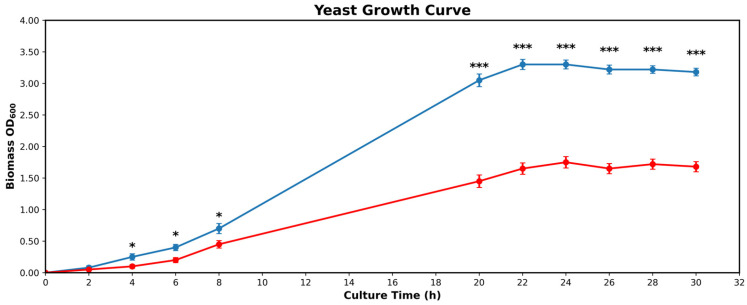
Growth Curve of ρ^+^ (blue) cultures reach stationary phase after 22 h. Growth Curve of ρ^0^ (red) cultures reach stationary phase after 24 h. Mean values (line) + SD (bars) of *n* = 3 biological replicates. Statistical significance was assessed by unpaired two-tailed *t*-test with Welch’s correction; * = *p* < 0.05; *** = *p* < 0.0005.

Prototrophic yeast strains were cultured in synthetic minimal (SM) medium consisting of 6.8 g/L Yeast Nitrogen Base without amino acids (YNB, DIFCO™) supplemented with 2% glucose (DIFCO™), unless otherwise specified. Growth rates of wild-type (ρ^+^) and respiratory-deficient yeast cells (ρ^0^) were determined by measuring cell density at OD600 in batch culture in SM medium (pH 5.0) for 30 h at 30 °C. The data reported for growth curves were the mean ± SD of three independent experiments. Growth rates were ρ^+^ = 0.1793 h^−1^ and ρ^0^ = 0.0861 h^−1^ (Figure 8).

For untargeted metabolomics experiments three independent biological replicates of ρ^+^ and ρ^0^ cells were grown in 10 mL SM medium to OD600 = 0.5 (~1 × 10^7^ cells). Metabolic activity was immediately quenched by the addition of cold methanol (*v*/*v*) at −80 °C. Biomass was collected by centrifugation (2 min, 4000× *g*, 4 °C).

### 4.3. Metabolite Extraction

Metabolites were extracted using a biphasic protocol adapted from established methods in the literature [22,31], with specific precautions to minimize artefacts and improve recovery. Cell pellets were resuspended in 200 μL of ACN:MeOH (75:25, *v*/*v*) containing 0.01 M ascorbic acid to stabilize molecules against oxidation [32,33] and disrupted with glass microspheres (500 nm) using a TissueLyser LT (QIAGEN, Hilden, Germany, 30 Hz, 6 min). After centrifugation (10 min, 13,000× *g*, 4 °C), the supernatant was collected. The pellet was subsequently treated with glass microspheres in TissueLyser LT (QIAGEN, 30 Hz, 6 min) in 200 μL of ultrapure water. The resulting lysate was centrifuged under the same conditions, and the aqueous supernatant was collected and combined with the organic fraction. Extracts corresponding to ~1 × 10^7^ yeast cells were dried in a vacuum concentrator (SpeedVac™ Savant™) and stored at −80 °C until LC–MS analysis.

Compared to classical extraction protocols employing boiling ethanol [34], which may induce unwanted chemical reactions (e.g., esterification or degradation of carboxylic acids), our approach avoids chemically aggressive conditions that could alter metabolite integrity. The use of glass beads and mechanical disruption ensures complete rupture of yeast cells, which possess a rigid cell wall not easily broken by mild treatments. This strategy allows the recovery of metabolites with different intrinsic lipophilicity while preserving their chemical nature.

Furthermore, cell washing has been reported as a critical step in yeast metabolomics [35,36,37]. Although washing may reduce metabolite yield, it improves signal-to-noise ratios and reduces background interference. In our workflow, cold methanol was used both to quench metabolism and to wash cells simultaneously, thereby balancing metabolite recovery with improved analytical quality.

Overall, this integrated extraction strategy combines the advantages of biphasic solvent partitioning, mechanical disruption, and antioxidant stabilization, providing a reproducible and chemically reliable method for untargeted metabolomics in yeast.

### 4.4. LC–MS Analysis

Dried extracts were reconstituted depending on the chromatographic mode. For hydrophilic interaction chromatography (HILIC), samples were resuspended in ACN:MeOH:Milli-Q water (70:20:10, *v*/*v*/*v*). For reversed phase (RP) C18 analysis, samples were dissolved in 7% ACN. Internal standards (3-acetyl-indole and N-acetyl-L-phenylalanine, 5 ppm) were added to all samples. After centrifugation (10 min, 13,000× *g*, 4 °C), supernatants were transferred to autosampler vials.

Metabolites were separated on a HILIC LC 150 Amide column (Thermo Fisher (Waltham, MA, USA), 100 × 2.1 mm, 5 μm) and a Hypersil C18 column (Thermo Fisher, 150 × 2.1 mm, 1.8 μm). Chromatography was performed at 30 °C (column temperature) with a flow rate of 300 μL min^−1^ using a Vanquish Flex UHPLC system (Thermo Scientific, Waltham, MA, USA). Detection was carried out on an Orbitrap Fusion™ Tribrid™ HRMS (Thermo Fisher Scientific) equipped with an electrospray ionization source.

RP C18 gradient (Eluents: A = water + 0.1% formic acid; B = ACN + 0.1% formic acid): 0–0.5 min, 2% B; 0.5–11 min, linear increase to 75% B; 11–13 min, 98% B; 18 min, return to 2% B; re-equilibration up to 25 min.

HILIC gradient (Eluents: A = 95% ACN, 5% water, 10 mM ammonium bicarbonate, 0.1% formic acid; B = 30% ACN, 70% water, 10 mM ammonium bicarbonate, 0.1% formic acid): 0–1 min, 1% B; 1–12 min, linear increase to 90% B; 12–14 min, 90% B; 14.5 min, return to 1% B; re-equilibration up to 21 min. Injection volume was 2 μL, with needle wash (MeOH:water, 1:1, *v*/*v*).

Two technical replicates of all samples were analyzed by both HILIC and RP separation in positive and negative ionization modes. Ionization potentials were set to +3.5/−3.3 kV, with ion transfer tube and vaporizer temperatures at 300 °C. Sheath gas flow was 40, and auxiliary gas flow was 8. MS^1^ spectra were acquired over a mass range of 50–1000 *m*/*z* at 120,000 resolution. MS^2^ spectra were acquired in data dependent scan at 15,000 resolution using higher-energy collisional dissociation (HCD, Orbitrap detection) setting Normalized Collision Energies (NCE) at 20, 40, 90. Low resolution MS^3^ spectra were acquired using collision-induced dissociation (CID) in the ion trap, with a NCE of 30%. The automatic gain control (AGC) target was set to 10,000 charges, with a maximum injection time of 35 ms (activation time: 10 ms; activation Q: 0.25), using the rapid scan mode. MS^2^ and MS^3^ precursor ions were isolated in the quadrupole and ion trap, respectively, using isolation windows of 1.8 Th for MS^2^ and 2.5 Th for MS^3^. MS^3^ acquisition was triggered in a Top 3 data dependent scan when MS^2^ fragment ion intensities exceeded a threshold of 5000 counts.

Samples were analyzed in randomized order, interspersed with blanks, pooled samples and CIL-QC. An integrated MS acquisition workflow (AcquireX, Xcalibur™ 4.2, Thermo Scientific™) was applied to improve data quality by prioritizing MS^n^ trigger of signals originating from biological samples and enabling deep interrogation of low-abundance species [13]. As an example of MS3 trigger efficiency, during the first identification (ID) injection of the AcquireX sequence using the Hilic column, approximately 900 MS^3^ spectra were acquired over a total run time of 21 min. These spectra were not uniformly distributed across the chromatographic run. Data acquisition was performed using a cycle time of 800 ms (corresponding to the interval between two MS^1^ scans), with chromatographic peaks exhibiting an average base width of approximately 8 s. The impact of MS^3^ acquisition on the reduction of MS^2^ triggering was not evaluated, as the iterative nature of injections during the identification step of a typical AcquireX workflow compensates the reduction in MS^2^ scan collection.

Raw data were processed using Compound Discoverer™ 3.3.0.5 (Thermo Fisher Scientific, Bremen, Germany) for untargeted metabolomics analysis. The parameters included for features detection are: 5 ppm mass tolerance, 0.2 min retention time tolerance, minimum peak intensity 100,000 a.u. Peak areas were normalized using cell number (OD600) of each sample and the internal standards run in each chromatographic sequence. Databases queried included KEGG, BioCyc, ChEBI, ChEMBL, LipidMAPS, and the Yeast Metabolome Database. Annotation was performed by retention time and MS^n^ spectral matching against an internal mzVault library and mzCloud.

### 4.5. Data Integration

For the automated integration of transcriptomics and metabolomics data, joint pathway analysis was performed using MetaboAnalyst 6.0. Official gene symbols and compound names, along with optional fold changes, were submitted to assess the potential importance of individual molecules according to their position within the network.

## 5. Conclusions

In summary, our work demonstrates that the combination of advanced chromatographic separation, high-resolution mass spectrometry, and complementary in silico tools can markedly improve annotation rates in untargeted metabolomics. This analytical strategy increased annotation coverage by ~4.5% through MS^3^-assisted fragmentation combined with FISh-based in silico scoring, which enabled discrimination of isobaric candidates and improved annotation confidence. Importantly, the differential metabolomics data analysis of ρ^0^ vs. ρ^+^ yeast cells was the basis for a data integration test with already published transcriptomic data of yeast lacking mtDNA in the same growth phase, allowing for pathway-level interpretation. MetaboAnalyst Joint Pathway Analysis module revealed coordinated changes between metabolites and their associated genes, enabling pathway-level interpretation and supporting the involvement of polyamine biosynthesis and aromatic amino acid metabolism in the metabolic adaptation of mtDNA-deficient yeast cells, thereby refining our understanding of mitochondrial dysfunction-driven metabolic reprogramming, which deserves future investigation.

Beyond yeast, these findings reinforce the relevance of model organisms in systems biology, where the critical mass of genetic and biochemical knowledge can accelerate methodological innovation and translational insights.

Future studies should focus on refining integrative multi-omics workflows through the development of species-specific genome-scale metabolic network models, overcoming limitations associated with KEGG-based generic pathway definitions and enabling more accurate biological interpretation, as well as improving computational annotation strategies to further resolve metabolic complexity and strengthen the connection between yeast systems biology and biomedical research.

## Figures and Tables

**Table 1:** Joint Pathway Analysis by MetaboAnalyst.

Pathways	−log10(*p*)	Impact ^1^
Aminoacyl-tRNA biosynthesis	6.2123	0.28986
Alanine, aspartate and glutamate metabolism	4.5611	1.1111
Arginine biosynthesis	3.6463	0.79412
Pyrimidine metabolism	3.0553	0.62821
Glutathione metabolism	2.5528	0.56522
Pantothenate and CoA biosynthesis	2.5155	0.4359
Monobactam biosynthesis	2.0885	0.6
One carbon pool by folate	1.74	0.35294
Valine, leucine and isoleucine biosynthesis	1.639	0.33333
Arginine and proline metabolism	1.604	0.44444
Lysine biosynthesis	1.5712	0.51724
Purine metabolism	1.4399	0.56618
Cyanoamino acid metabolism	1.3167	0.35294

^1^ The pathway impact is calculated as the sum of the importance measures of the matched metabolites normalized by the sum of the importance measures of all metabolites in each pathway.

## Data Availability

The data generated during this study are included within the article in Supplementary Data files. Raw metabolomic data have been deposited to MetaboLights [38] repository with the study identifier MTBLS13540 (https://www.ebi.ac.uk/metabolights/MTBLS13540, accessed on 22 January 2026).

## Supplementary Materials

The following supporting information can be downloaded at: https://www.mdpi.com/article/10.3390/ijms27062624/s1.

## Abbreviations

The following abbreviations are used in this manuscript:

PCA	Principal Component Analysis
UHPLC	Ultra-High-Performance Liquid Chromatography
HRMS	High-Resolution Mass Spectrometry
RT	Retention Time
FISh	Fragment Ion Search
