# Supplementary material for: Enhanced Untargeted Metabolomics Based on High-Resolution Mass Spectrometry Reveals Global Rewiring Due to Mitochondrial Dysfunction in Yeast
Source: Int J Mol Sci. 2026 Mar 13;27(6):2624. doi: 10.3390/ijms27062624 (PMC13026841; doi:10.3390/ijms27062624)
Supplement: Supplementary file 1 [file ijms-27-02624-s001.zip › Figure S1&Figure S2.pdf]

Name	MW	STD Intensity Blank	STD Intensity 1:10	STD Intensity TQ
Alanine	89.0476	1.04E+05	1.29E+07	1.49E+08
Adenosine	267.0966	1.10E+04	1.51E+06	8.65E+06
Guanine	151.0494	1.02E+05	1.53E+08	1.33E+09
Guanosine	283.0916	2.11E+04	2.67E+07	1.59E+08
Malate	134.0215	1.31E+04	8.19E+05	4.12E+06
Threonine	119.0582	1.43E+04	1.18E+07	1.43E+08
		STD Conc. Blank	STD Conc. 1:10 (mM)	STD Conc. TQ (mM)
		0.00E+00	2.25	22.5
		0.00E+00	0.75	7.5
		0.00E+00	1.32	13.2
		0.00E+00	0.71	7.1
		0.00E+00	1.49	14.9
		0.00E+00	1.68	16.8

	Rho 0	Rho +	Rho 0 conc.mM	Rho + conc.mM
Alanine	8.71E+06	8.87E+06	1.02	1.03
Adenosine	2.01E+08	3.32E+08	180.67	298.87
Guanine	1.42E+07	2.39E+07	0.04	0.14
Guanosine	8.05E+06	1.37E+07	0.17	0.45
Malate	3.88E+07	3.84E+07	154.47	152.87
Threonine	8.21E+06	1.38E+07	0.96	1.52

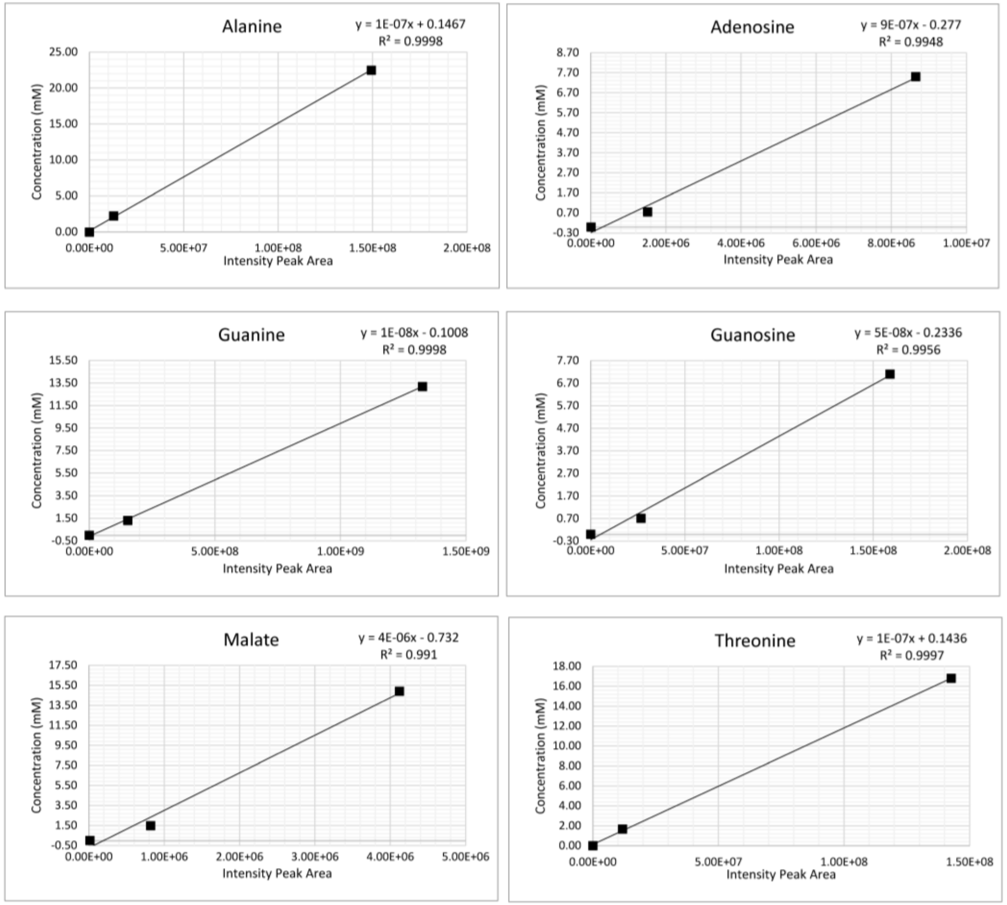

Figure S1. Calibration curves and metabolite quantification. Linear calibration curves (peak area vs. concentration, mM) are shown for alanine, adenosine, guanine, guanosine, malate, and threonine; the regression equation and coefficient of determination (R^2) are reported within each panel. The upper tables list name of each standard (STD), molecular weight (MW), peak areas for Blank, 1:10 diluted STD and neat standard (TQ), together with the corresponding standard concentrations (mM). The lower table summarizes measured peak areas and calculated concentrations (mM) for two experimental conditions (Rho0 and Rho+). All calibrations were linear across the tested ranges. Abbreviations: MW, molecular weight; STD, standard; TQ, neat standard; Rho0, ϱ0; Rho+, ϱ+.

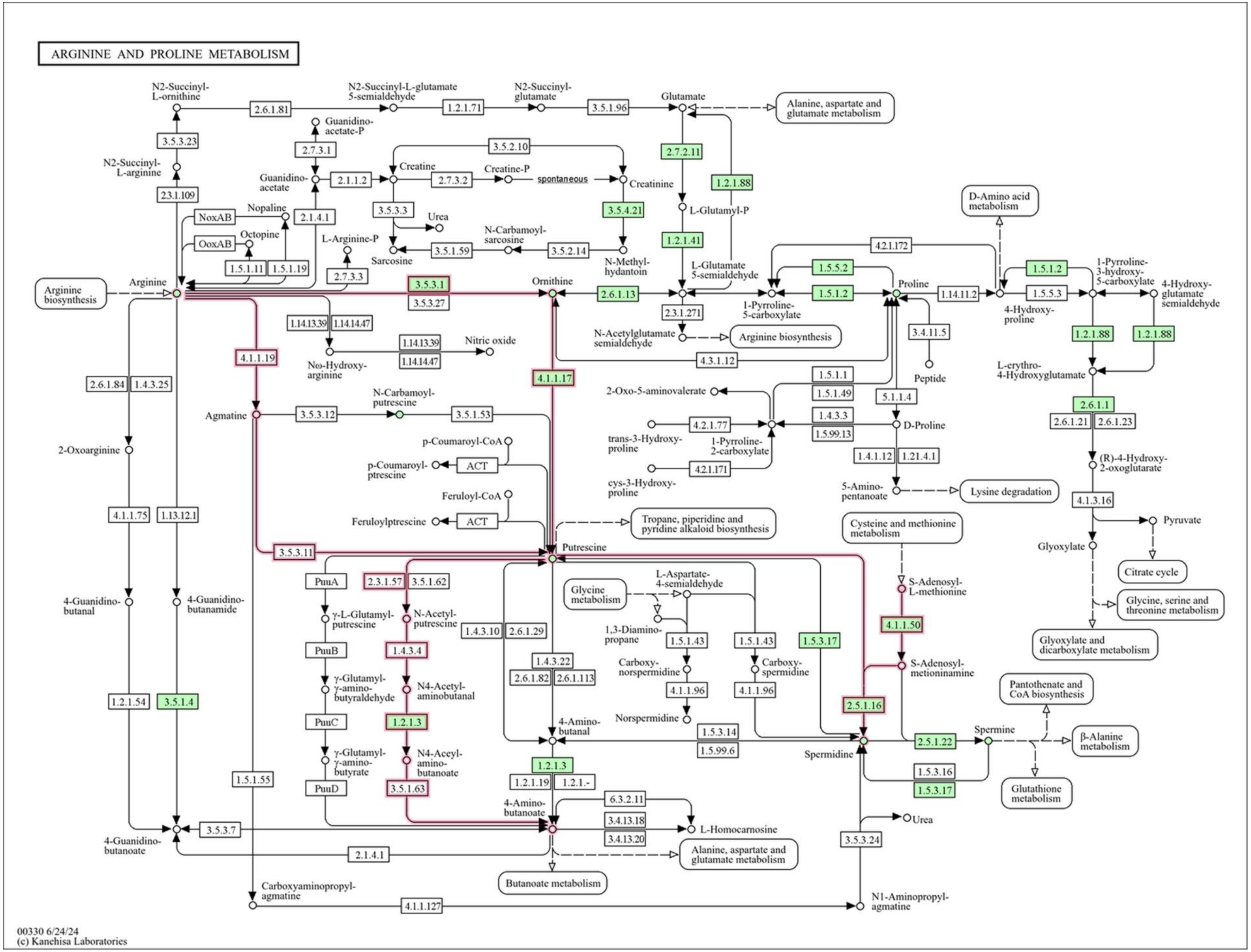
ARGININE AND PROLINE METABOLISM
N2-Succinyl-L-ornithine
2.6.1.81
N2-Succinyl-L-glutamate 5-semialdehyde
1.2.1.71
N2-Succinyl-glutamate
3.5.1.96
Glutamate
Alanine, aspartate and glutamate metabolism
3.5.3.23
N2-Succinyl-L-arginine
2.3.1.109
Guanidino-acetate-P
2.7.3.1
Guanidino-acetate
2.1.1.2
Creatine
2.7.3.2
Creatine-P
spontaneous
Creatinine
3.5.2.10
3.5.3.3
Urea
3.5.4.21
2.7.2.11
1.2.1.88
L-Glutamyl-P
1.2.1.41
D-Amino acid metabolism
NoxAB
Nopaline
2.1.4.1
OoxAB
Octopine
L-Arginine-P
N-Carbamoyl-sarcosine
3.5.1.59
3.5.2.14
Sarcosine
N-Methyl-hydantoin
1.5.1.11
1.5.1.19
2.7.3.3
L-Glutamate 5-semialdehyde
4.2.1.172
1.5.5.2
1.5.1.2
1-Pyrroline-3-hydroxy-5-carboxylate
4-Hydroxy-glutamate semialdehyde
Arginine biosynthesis
Arginine
3.5.3.1
3.5.3.27
Ornithine
2.6.1.13
2.3.1.271
1-Pyrroline-5-carboxylate
Proline
1.14.11.2
1.5.5.3
4-Hydroxy-proline
N-Acetylglutamate semialdehyde
Arginine biosynthesis
3.4.11.5
1.14.13.39
1.14.14.47
Nitric oxide
Nω-Hydroxy-arginine
4.3.1.12
Peptide
4.1.1.19
4.1.1.17
1.2.1.88
L-erythro-4-Hydroxyglutamate
2.6.1.84
1.4.3.25
2-Oxo-5-aminovalerate
1.5.1.1
1.5.1.49
5.1.1.4
2.6.1.1
2.6.1.21
2.6.1.23
Agmatine
3.5.3.12
N-Carbamoyl-putrescine
3.5.1.53
4.2.1.77
1.4.3.3
1.5.99.13
D-Proline
trans-3-Hydroxy-proline
1-Pyrroline-2-carboxylate
p-Coumaroyl-CoA
2-Oxoarginine
p-Coumaroyl-ptrescine
ACT
4.2.1.171
1.4.1.12
1.21.4.1
(R)-4-Hydroxy-2-oxoglutarate
cys-3-Hydroxy-proline
5-Amino-pentanoate
Lysine degradation
Feruloyl-CoA
4.1.3.16
4.1.1.75
1.13.12.1
Feruloylptrescine
Tropane, piperidine and pyridine alkaloid biosynthesis
Cysteine and methionine metabolism
Pyruvate
Glyoxylate
3.5.3.11
Putrescine
Citrate cycle
PuuA
2.3.1.57
3.5.1.62
L-Aspartate-4-semialdehyde
S-Adenosyl-L-methionine
4-Guanidino-butanal
4-Guanidino-butanamide
γ-L-Glutamyl-putrescine
N-Acetyl-putrescine
Glycine metabolism
Glycine, serine and threonine metabolism
1.4.3.10
2.6.1.29
1,3-Diamino-propane
4.1.1.50
PuuB
1.4.3.4
1.5.1.43
1.5.1.43
1.5.3.17
Glyoxylate and dicarboxylate metabolism
1.4.3.22
γ-Glutamyl-γ-amino-butyraldehyde
N4-Acetyl-aminobutanal
2.6.1.82
2.6.1.113
Carboxy-norspermidine
Carboxy-spermidine
S-Adenosyl-methioninamine
Pantothenate and CoA biosynthesis
1.2.1.54
3.5.1.4
4.1.1.96
4.1.1.96
2.5.1.16
PuuC
1.2.1.3
Norspermidine
4-Amino-butanal
1.5.3.14
Spermine
β-Alanine metabolism
γ-Glutamyl-γ-amino-butyrate
N4-Acetyl-amino-butanoate
1.5.99.6
Spermidine
2.5.1.22
1.2.1.3
1.5.3.16
1.5.1.55
PuuD
3.5.1.63
1.2.1.19
1.2.1.-
1.5.3.17
Glutathione metabolism
4-Amino-butanoate
6.3.2.11
3.4.13.18
L-Homocarnosine
Urea
3.5.3.7
3.4.13.20
4-Guanidino-butanoate
2.1.4.1
Alanine, aspartate and glutamate metabolism
3.5.3.24
Butanoate metabolism
Carboxyaminopropyl-agmatine
N1-Aminopropyl-agmatine
4.1.1.127
00330 6/24/24
(c) Kanehisa Laboratories

Figure S2. Joint Pathway Analysis of the polyamine pathway within the arginine and proline metabolism. The network shows metabolites and enzymes involved in polyamine biosynthesis, mapped onto KEGG-based pathway definitions using MetaboAnalyst 6.0. Red lines highlight the polyamine pathway within the arginine and proline metabolism, while green boxes indicate genes and metabolites identified as differentially expressed within this pathway.
